# Supplementary material for: Molecular Modeling and Gene Ontology Implicate SLC35F4 and SLC35F5 as Golgi-Associated Importers of Flavin-Adenine-Dinucleotide
Source: Int J Mol Sci. 2026 Jan 4;27(1):512. doi: 10.3390/ijms27010512 (PMC12786601; doi:10.3390/ijms27010512)
Supplement: Supplementary file 1 [file ijms-27-00512-s001.zip › ijms-4033556-supplementary.pdf]

Supplementary Materials

Zheyun N, Jiang D, Hardy DM 2025 Int J Mol Sci

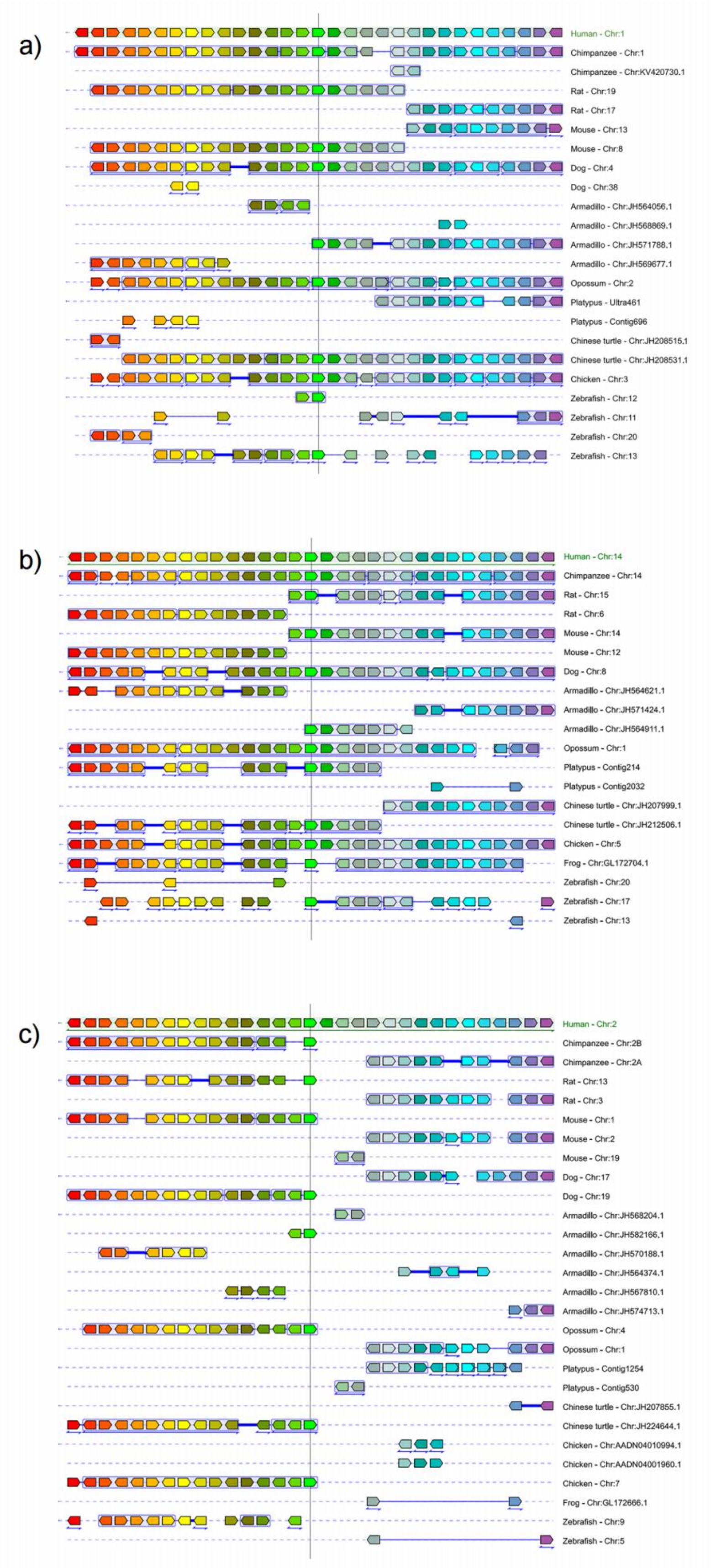

Figure S1. Shared synteny of SLC35F family genes across representative vertebrate genomes. a) *SLC35F3* synteny plot comparing gene order and orientation (colored, directional arrows) of the genomic locus spanning 15 genes on each side of the human gene among 11 vertebrate species. Note the extensive shared synteny, highlighting the evolutionary stability of the *SLC35F3* locus. b) *SLC35F4* comparative synteny plot. Note the relative evolutionary stability of the *SLC35F4* locus. c) *SLC35F5* comparative synteny plot. Note the shared synteny among mammals, reptiles, and amphibians, but absence of *SLC35F5* in *Danio rerio*. Plots were generated using the Genomicus v93.01 AlignView tool (reference species: human; root species: Euteleostomi, ~420 million years), with gene names and chromosomal positions were obtained from the integrated Ensembl annotations within Genomicus.

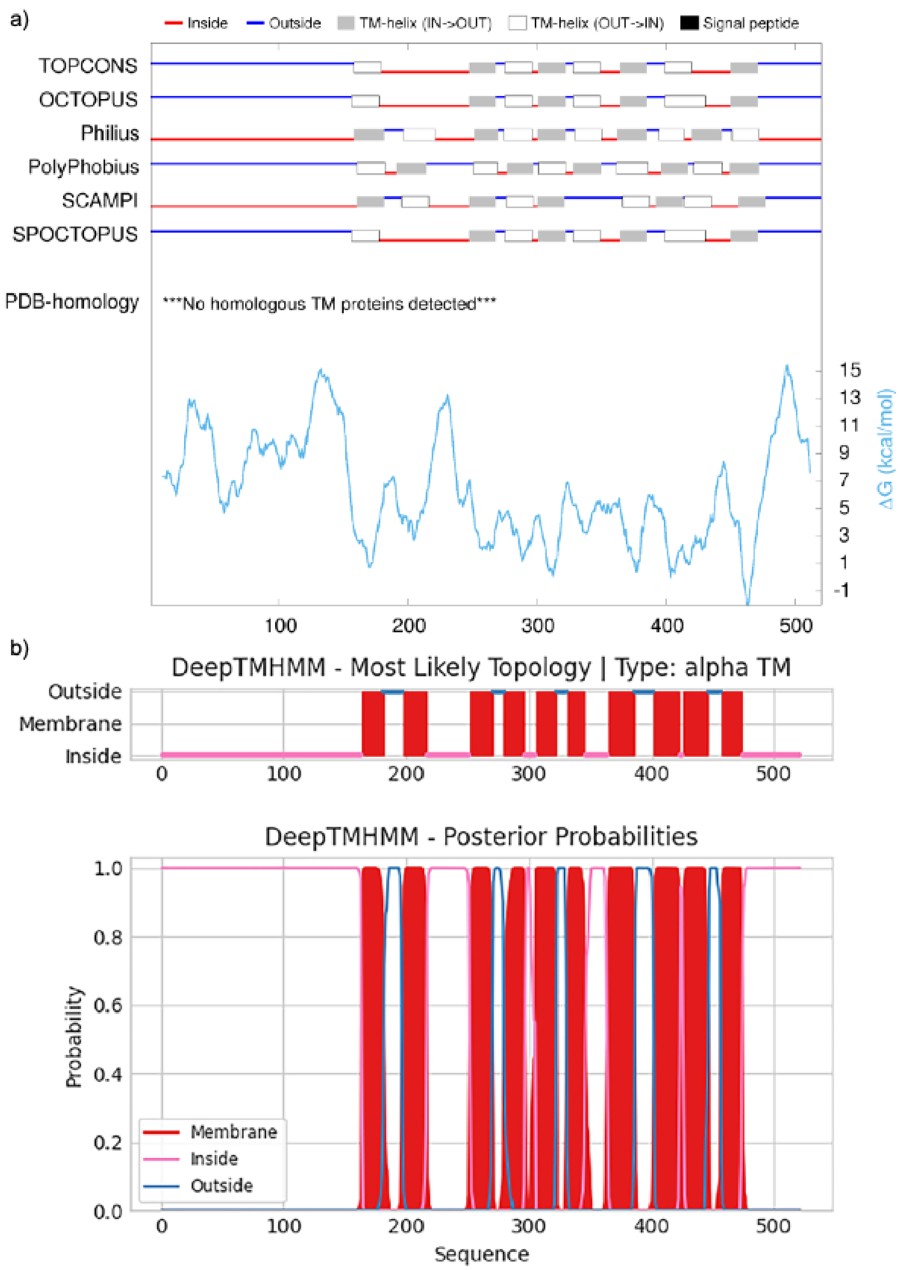

Figure S2. Predicted membrane insertion energy and transmembrane topology for SLC35F4. a) Integrated transmembrane topology predictions generated by the TOPCONS platform for SLC35F4. Each row represents a different prediction algorithm (TOPCONS, OCTOPUS, Philius, PolyPhobius, SCAMPI, SPOCTOPUS). Red and blue lines indicate predicted cytoplasmic and extracellular regions, respectively. Gray and white boxes indicate transmembrane helices oriented from cytoplasm to outside (IN→OUT) or outside to cytoplasm (OUT→IN). Black boxes indicate predicted signal peptides, if present. The Predicted membrane insertion free energy (ΔG, kcal/mol) plotted along the amino acid sequence. The x-axis indicates residue position, while the y-axis represents the estimated energetic cost for membrane insertion at each position. b) DeepTMHMM-based topology prediction for SLC35F4. The top panel shows the most likely topology with color-coded membrane (red), cytoplasmic (pink), and extracellular (blue) segments along the protein sequence. The bottom panel shows probabilities of each position being in membrane, inside, or outside environments, with y-axis indicating confidence levels (0 to 1.0).

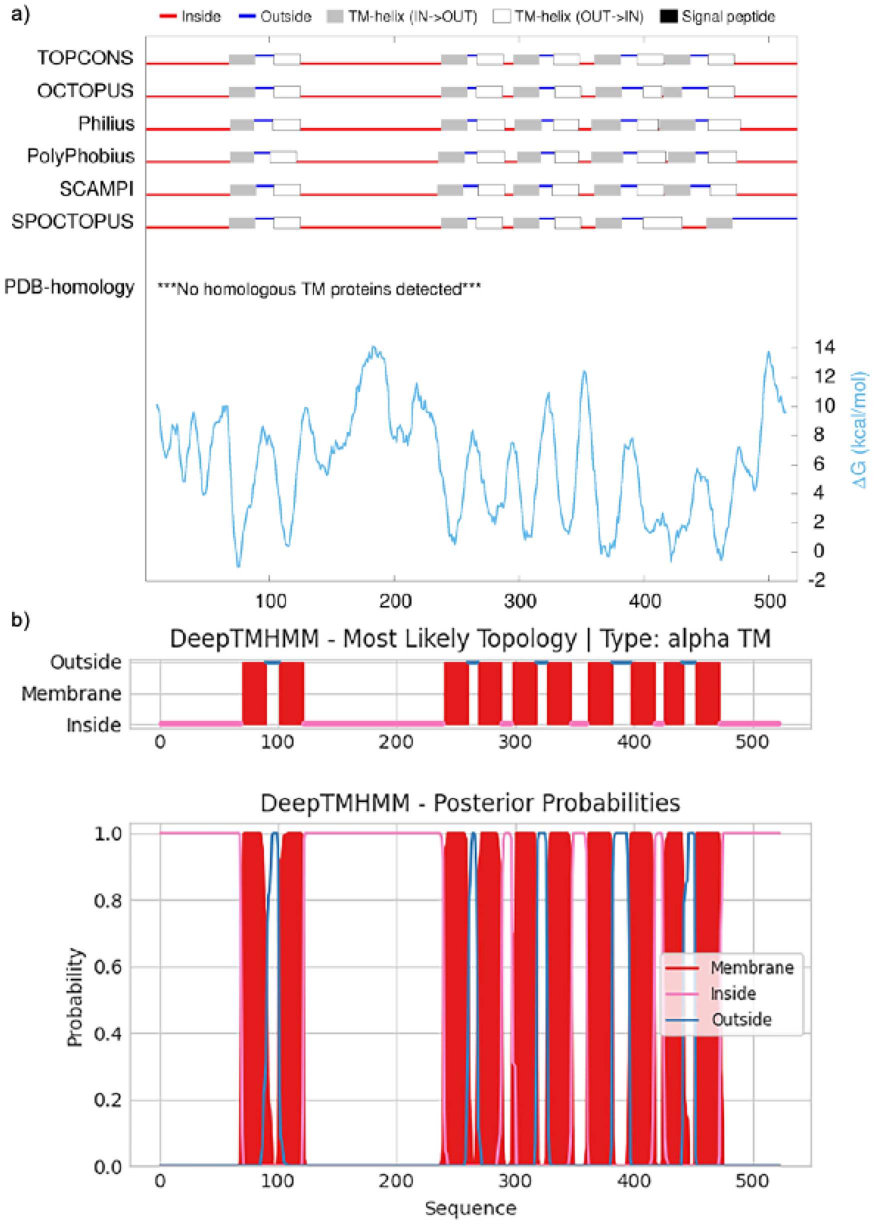

Figure S3. Predicted membrane insertion energy and transmembrane topology for SLC35F5. a) Integrated transmembrane topology predictions generated by the TOPCONS platform for SLC35F5. Each row represents a different prediction algorithm (TOPCONS, OCTOPUS, Philius, PolyPhobius, SCAMPI, SPOCTOPUS). Red and blue lines indicate predicted cytoplasmic and extracellular regions, respectively. Gray and white boxes indicate transmembrane helices oriented from cytoplasm to outside (IN→OUT) or outside to cytoplasm (OUT→IN). Black boxes indicate predicted signal peptides, if present. The Predicted membrane insertion free energy (ΔG, kcal/mol) plotted along the amino acid sequence. The x-axis indicates residue position, while the y-axis represents the estimated energetic cost for membrane insertion at each position. b) DeepTMHMM-based topology prediction for SLC35F5. The top panel shows the most likely topology with color-coded membrane (red), cytoplasmic (pink), and extracellular (blue) segments along the protein sequence. The bottom panel shows probabilities of each position being in membrane, inside, or outside environments, with y-axis indicating confidence levels (0 to 1.0).

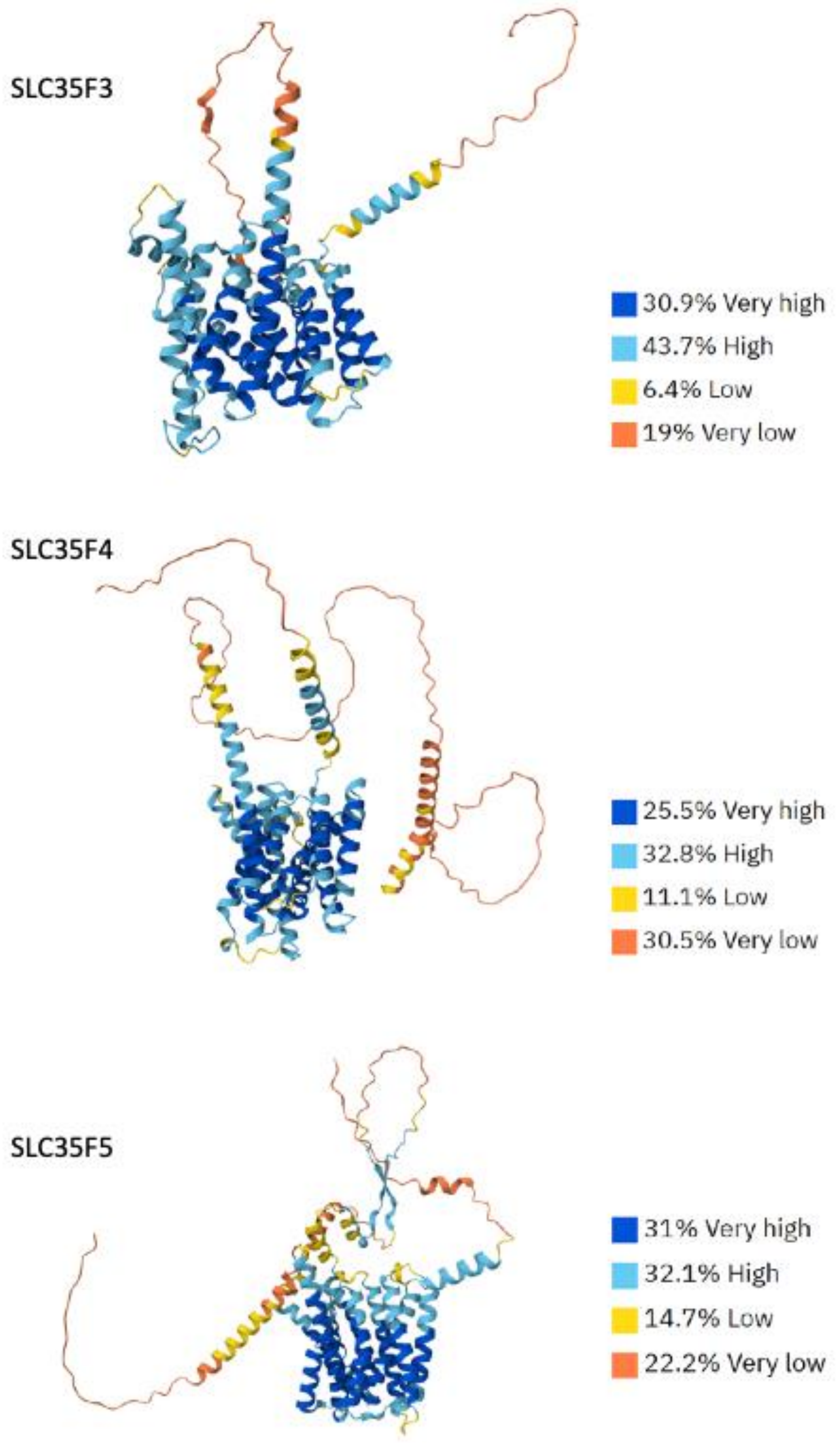

Figure S4. Predicted high and low confidence regions of SLC35F3-F5 indicated by pLDDT statistics from the AlphaFold3 model used for functional annotation. Colors denote regions with Very High, High, Low, and Very Low confidence. Note the consistently High or Very High confidence in the functionally important transmembrane segments.
